# Draft Genome Assembly of the Bloom-Forming Cyanobacterium *Nodularia spumigena* Strain CENA596 in Shrimp Production Ponds

**DOI:** 10.1128/genomeA.00466-16

**Published:** 2016-06-09

**Authors:** Rafael Vicentini Popin, Janaina Rigonato, Vinicius Augusto Carvalho Abreu, Ana Paula Dini Andreote, Savênia Bonoto Silveira, Clarisse Odebrecht, Marli Fatima Fiore

**Affiliations:** a University of São Paulo, Center for Nuclear Energy in Agriculture, Piracicaba, São Paulo, Brazil; b Federal University of Rio Grande, Institute of Oceanography, Rio Grande, Rio Grande do Sul, Brazil

## Abstract

We report here the draft genome assembly of the brackish cyanobacterium *Nodularia spumigena* strain CENA596 isolated from a shrimp production pond in Rio Grande do Sul, Brazil. The draft genome consists of 291 contigs with a total size of 5,189,679 bp. Secondary metabolite annotations resulted in several predicted gene clusters, including those responsible for encoding the hepatotoxin nodularin.

## GENOME ANNOUNCEMENT

*Nodularia spumigena* is a cyanobacterium species known for producing the hepatotoxin nodularin (1), a cyclic pentapeptide with a chemical structure similar to microcystin. Nodularin also acts as an inhibitor of the protein phosphatase serine/threonine family, particularly phosphatases type 1 (PP1) and 2A (PP2A) of eukaryotic cells (2–4), and it is a suspected carcinogen and tumor promoter (5). Due to the persistence of nodularin in the environment and possible processes of bio-accumulation in organisms, environmental and economic problems as well as harm to animals and humans may occur in areas with cyanobacterial bloom. The occurrence of major blooms of the halotolerant species *N. spumigena* has been described in ocean coastlines, estuaries, and saline lakes of Europe, Australia, New Zealand, and the African continent (6–12). In the American continent, the presence of *N. spumigena* bloom was reported in saline lakes and ponds in the United States (13, 14), Mexico (15), and Uruguay (16). The first report of *N. spumigena* documented in Brazil was in 2011 in shrimp production ponds, causing the death of these crustaceans (17). Here, we announce the draft genome assembly of the strain CENA596 isolated from an *N. spumigena* bloom in 5 December 2013 in a shrimp production pond (32°12′19″ S, 52°10′42″ W) in the Marine Aquaculture Station of the Federal University of Rio Grande, located on the Cassino Beach, Rio Grande, Rio Grande do Sul State, Brazil. Genomic DNA extraction was performed using the UltraClean microbial DNA isolation kit (MoBio Laboratories, USA) and quantified using the Qubit Fluorometer (Thermofisher/Life Technology, USA). Whole genome sequencing was performed with MiSeq platform (Illumina, USA) using the MiSeq Reagent Kit v3 600 cycle (Illumina). Bases with quality scores under Phred 20 and sequences shorter than 100 bp were removed with PRINSEQ 0.20.4 (18). *De novo* genome assembly was carried out with *Platanus* 1.2.4 (19). Assembly statistics were obtained with Assemblathon 2 (20). The final draft genome assembly consisted of 699-fold average coverage and 291 contigs (>526-bp length), with a total size of 5,189,679 bp, and GC content of 41.2%. The automatic annotation using Prokka 1.10 (21) predicted 4,484 coding sequences, 36 tRNA genes, and 1 rRNA gene. The RAST annotation system (22) predicted 370 subsystems, which represent only 33% of the assigned sequences. Genes encoding resistance to heat shock, osmotic and oxidative stress, antibiotics, toxic compounds such as mercury, copper, and chromium, along with genes involved in nitrogen metabolism, siderophores, and auxin biosynthesis were found. Secondary metabolite gene cluster prediction was done using the antiSMASH 3.0 server (23) and resulted in 12 predicted gene clusters indicating the potential biosynthesis of nodularin, spumigin, anabaenopeptin, and geosmin, among others. This *Nodularia spumigena* genome sequence from South America contributes to the improvement of the current classification of the halotolerant cyanobacterial group and to the understanding of the genetic, ecological, and evolutionary factors related to nodularin production, which is important information for the development of rapid and sensitive methods for the detection and monitoring of cyanobacteria and their toxins in the environment.

### Nucleotide sequence accession number.

The *Nodularia spumigena* CENA596 whole-genome shotgun project has been deposited at DDBJ/EMBL/GenBank under the accession number LWAJ00000000. The version described in this paper is version LWAJ01000000.
